# Evaluating the implementation of a mental health joint response with young people and families: protocol for the care responders study, a realist and health economic evaluation

**DOI:** 10.3389/frhs.2026.1846383

**Published:** 2026-06-26

**Authors:** Sarah Parry, Zarah Eve, Heather Brown, Fiona Lobban, Prathiba Chitsabesan, Debbie Robinson, Karina Lovell, Adele Terry, Kathryn Harper, Kelly Brodie, Geoff Wong

**Affiliations:** 1 Faculty of Biology, Medicine and Health, University of Manchester, Manchester, United Kingdom; 2 Pennine Care NHS Foundation Trust, 225 Old Street, Ashton-under-Lyne, Lancashire, United Kingdom; 3 Faculty of Health and Education, School of Nursing and Public Health, Manchester Metropolitan University, Manchester, United Kingdom; 4 Faculty of Health and Medicine, Division of Health Research, Lancaster University, Lancaster, United Kingdom; 5 Greater Manchester Mental Health NHS Foundation Trust, Manchester, United Kingdom; 6 Nuffield Department of Primary Care Health Sciences, Medical Sciences Division, The University of Oxford, Oxford, United Kingdom

**Keywords:** children, co-response, crisis, emergency responder, mental health, protocol, young people

## Abstract

**Introduction:**

Mental health crises among children and young people are increasing in frequency and complexity, yet emergency responses often lack appropriate tailoring for young people and responders cite a need for further workforce training and support. This study will integrate immersive theatre, co-production methods, health economic analysis, research data, and a realist synthesis to develop a holistic programme theory informing joint responses to crisis care for children and young people. It will incorporate novel triangulation across these methods and, where appropriate, draw on the recently published Realist Economic Evaluation Methods(REEMS) guidance.

**Methods:**

This study aims to evaluate a novel intervention involving a joint response by police officers and mental health practitioners. A realist and health economic evaluation design will be employed, incorporating multiple data collection methods. Participants will include children and young people aged 5–18, their families, carers, and practitioners. Data will be collected through surveys, interviews, and routine service records, with recruitment via five distinct pathways including direct response and online participation.

**Results:**

The evaluation will explore how, why, and for whom the joint response car works, identifying mechanisms and contextual factors that influence outcomes. A cost-consequence analysis will assess the financial implications of the intervention compared to usual care.

**Discussion:**

Findings will inform best practice guidance for emergency mental health care and support national implementation of joint response models. The study will also contribute to understanding service integration and stakeholder engagement in crisis care.

## 1 Introduction

Historically, mental health crises have often been managed through emergency departments or acute psychiatric services, which are often adult-focused and not equipped to provide developmentally appropriate or coordinated responses for children and young people (CYP) (1). Mental health crisis services for children and young people in England and Wales have recently been found to comprise a diverse mix of provision, most commonly community in-person rapid response teams that deliver assessment and support in homes, schools, or other community settings. Joint and co-responses are also increasing in popularity and include models such as emergency mobile responses (e.g., police or ambulance-linked services), crisis assessment and triage joint responses (e.g., mental health practitioner and first responder model), and intensive home treatment as an alternative to hospital admission. Hospital-based services, particularly emergency departments that have seen increases in youth admissions (2), continue to play a key role when risk is high, alongside specialist CAMHS crisis teams providing short-term intervention. In addition, crisis support may be delivered through helplines, digital services, and non-clinical provision such as schools and voluntary sector organisations, reflecting a highly variable and fragmented system of care (3). Inconsistent care provision for children and young people experiencing a mental health crisis can mean highly vulnerable children do not have access to timely and appropriate care, which can cause further distress for the young person family, and first responders trying to offer support.

International guidance emphasises a tiered, child-centred approach to supporting children and young people in mental health crises, combining early interventions such as Psychological First Aid with evidence-based therapies, while prioritising safety, rights (4, 5), and the prevention of re-traumatisation across community and specialist care systems. Importantly, trauma-responsive, formulation-led approaches are increasingly recognized as helpful to promote stabilisation, trusting relationships, and collaborative, individualised planning to address the child's underlying needs and promote recovery (6). However, there remains a substantial global gap in policies for child and adolescent mental health, especially tailored for a mental health crisis. In 2004, Shatkin et al (7)., found only 35 countries (18%) had policies including provisions for young people, and none were exclusively dedicated to this group. Although policy development has progressed, considerable variation persists in approaches to service delivery, workforce development, research, and public education. Dedicated policies are both feasible and critical for strengthening service systems, improving data collection, promoting culturally responsive care, and ensuring sustainable funding. The COVID-19 pandemic exposed and intensified existing vulnerabilities in child and adolescent mental health and services to support them, with evidence of increased emotional and behavioural difficulties, self-harm, suicidality, and disruption to education, relationships, and service access. Intervention for young people experiencing a mental health crisis needs to be expanded in terms of access to evidence-based support and more resilient systems capable of responding to future crises (8). Some broader frameworks provide guiding principles. For example, Mental Health and Psychosocial Support (MHPSS) (9) refers to a range of interventions that promote psychological wellbeing, strengthen social connections, and support mental health. When supporting children and young people through a mental health crisis, MHPSS is guided by principles of safety, trust, dignity, participation, and relationship-based care. It emphasises understanding distress within the context of a young person's experiences, relationships, family, culture, and environment, rather than focusing solely on symptoms. Effective MHPSS responses seek to reduce immediate distress, strengthen existing sources of support, promote hope and resilience, and ensure that children and young people are actively involved in decisions about their care and recovery. Further, evidence from humanitarian settings suggests that while structured psychological therapies can improve outcomes for young people, intervention effects are highly context-dependent and, in some cases, may be adverse, highlighting the importance of careful adaptation and evaluation when scaling mental health provision in different systems (10), such as the NHS.

Systems achieve better outcomes for children and young people when they work together (11). This has prompted interest in integrated crisis care systems that aim to provide timely and age-appropriate assessment and crisis support. In the UK, clinical guidance for practitioners responding to children and young people experiencing a mental health crisis is primarily provided by the National Institute for Health and Care Excellence (*N*ICE), such as guidance on self-harm (NG225) and psychosis (CG155). This guidance emphasises the need for rapid access to specialist assessment, often within hours for acute presentations, and advocate for care to be delivered by appropriately trained professionals within child and adolescent mental health services. These guidelines prioritise comprehensive psychosocial assessment, collaborative safety and crisis planning, and the involvement of families or carers, while promoting the least restrictive approach by supporting young people in community or home settings wherever it is safe to do so. They also highlight the importance of coordinated aftercare, continuity between services, and attention to safeguarding needs. Complementary guidance from NHS England further supports implementation by outlining expectations for 24/7 crisis response, integrated care pathways, and timely access to support across emergency, community, and inpatient settings. However, out of hours care is rarely available for children and young people, which means that first responders are often without access to the means to provide the care recommended.

Joint response models have been implemented in some settings to support people experiencing mental health crises when emergency services are involved, which is increasingly likely during evenings and weekends when children's services are generally not available. These models commonly involve collaboration between police or paramedics and mental health professionals, with the aim of improving crisis assessment and facilitating access to appropriate mental health support (12, 13). In addition to crisis response services, collaborative approaches such as joint crisis planning have been proposed to support communication and shared decision-making between service users, families and professionals in managing and preventing crises (14). These approaches are typically found to be welcomed by families, as well as first responders. For example, police officers frequently feel ill-equipped to provide appropriate mental health support, particularly when responding to children and young people in crisis (15).

Most of the available literature on crisis response models originates from high-income countries, particularly the United States and the United Kingdom, and evidence specific to CYP remains limited (1, 16, 17). As a result, there is a approaches can be implemented and evaluated for children need for further research to examine how collaborative crisis response and young people.

In the UK, children and young people are experiencing escalating challenges to their mental health, with services “constantly firefighting” to meet demand(18). Pathways to mental health care for young people remain under-researched(19) and poorly understood. In February 2023, it was reported that the National Health Service (NHS) was not meeting its targets to improve access to and reduce waiting times for young people's mental health services (20). There is a pressing need for innovative and evidence-based approaches to support children and young people in crisis. In 2022, Greater Manchester Police (GMP) and three Greater Manchester Trusts piloted a Mental Health Joint Response Car (MHJRC) for adults across the city.

The MHJRC brings together a police officer and a mental health practitioner to respond collaboratively to individuals contacting emergency services in crisis. This model combines the rapid response capability of the police with the specialist expertise of mental health professionals. Early evidence demonstrates that joint responses can reduce unnecessary detentions under the UK's Mental Health Act, avoid hospital admissions, strengthen cross-sector relationships, increase user engagement, and reduce costs to public services (12, 21). The 2022 Greater Manchester pilot supported 1,484 adult patients. Outcomes included 673 avoided Emergency Department (ED) referrals and 521 avoided detentions under Section 136 (S136) of the Mental Health Act (S136 detentions enable the police to take a person to health facility for a mental health assessment when they suspect this may be present). Where S136 detentions were necessary, MHJRC involvement enhanced the pathway, improving patient experience. These results suggest the MHJRC model has significant potential to reduce inappropriate and distressing interventions whilst delivering proportionate, timely care.

Young people's mental health difficulties have intensified in scale and complexity in recent years, with COVID-related school closures and social media-related harms cited as contributory factors (22). Emergency demand (e.g., 999 calls, S136 and ED attendance) has increased, yet little is known about what effective emergency support for young people, and their families should look like. For example, GMP data indicate that S136 detentions of under-18s rose from 77 cases in 2020–21 to 115 cases in 2021–22. Localised British Transport Police data also indicates a sharp increase in emergency responses to CYP during school term time. Despite the clear need, existing emergency care provision for young people is extremely limited and often perceived as unsupportive. A survey of service users found that 54% rated child and adolescent mental health service (CAMHS) emergency care as ‘poor' or ‘awful', 40% believed service quality had worsened in the past three years, 64% reported no access to care after 17:00, and two-thirds experienced waiting times of more than 24 h for a hospital bed (23).

Current CAMHS provision largely operates within standard working hours (08:00–18:00), making integration with emergency services challenging. As a result, police and ambulance services are often first responders, despite evidence that police and ambulance services may lack appropriate training to respond to children experiencing a mental health crisis, and that stigma and insensitive practice can influence care (24). Importantly, existing data on mental health crisis calls is not routinely disaggregated by age, leaving a critical gap in understanding the needs of children and young people (25). Effective, proportionate on-scene intervention can reduce distress, avoid restrictive practices, and improve both immediate and longer-term outcomes. Current data suggest that most young people detained under S136 or taken to hospital by the police are not subsequently admitted (PCFT, internal data), raising concerns about the appropriateness of current pathways. MHJRCs could offer a more therapeutic and proportionate alternative, with potential to reduce unnecessary referrals, minimise trauma, and generate cost savings, given that a CAMHS hospital bed in the UK costs around £700 per person per day.

The proposed intervention for pilot and evaluation is of a MHJRC specifically for children aged 5 to 18 years of age, which we have called the Care Responders service. It is novel but builds upon a theoretically informed and co-produced adult model. Risks are anticipated to be lower than current practice, which is variable and often inappropriate. Delivery will be supported by a dedicated CAMHS practitioners and a highly trained research team, ensuring high standards of quality and participant safety. This protocol provides a detailed account of a collaborative realist approach to understanding the impact in real-world settings of a complex intervention, which will enhance transparency and inform the ongoing refinement of realist methodology in child and adolescent mental health research.

## 2 Method

### 2.1 Aims

1. Develop a programme theory of the impacts (positive and negative) of a Care Responders service for young people experiencing a mental health crisis and their families (workstream 1).
2. Draw upon the programme theory, lived experience, and realist evaluation to critically consider how a Care Responders service can integrate into existing service infrastructures to best serve young people in crisis (workstreams 2 and 3).
3. Identify the costs and effects of the Care Responders service for young people, their families, professionals, and systems (workstream 3).
4. Use the programme theory and evaluation outcomes to develop best practice tools for implementation and identify opportunities for integrating the Care Responders service within young people's mental health and social care services (workstream 4).

### 2.2 Objectives

1. Develop theories of the underlying generative mechanisms by which, and contexts within which, a joint response between a police officer and mental health practitioner impact on mental health, options for care, and wellbeing outcomes for young people in mental health crisis.
2. Develop a theory to understand the roles of police officers and mental health practitioners, how they vary in different contexts across call outs, and how they impact young people across childhood, adolescence, and emerging adulthood.
3. Test and refine the theories through qualitative enquiry with young people and their families, police officers, mental health practitioners, and other connected first responders.
4. Employ a cost-consequence approach to identify multiple effects across different sectors of the Care Responders service and compare with the costs of the intervention.
5. Test and refine the theories through qualitative enquiry with young people, parents/carers, practitioners, and wider stakeholders (e.g., commissioners).
6. Co-design effective outputs to share new learning about a Care Responders service for young people and engage national stakeholders to carry recommendations forwards.

### 2.3 Design

This study adopts a realist evaluation design with multiple data collection methods to assess the Care Responders pilot for young people experiencing a mental health crisis. The overarching initial programme theory that will be tested and refined throughout the study is “if we combine the speed of the police and the expertise of mental health practitioners from children and young people's services, they together could provide an optimal response to CYP and parent-caregivers for CYP in mental health crisis.” This was developed from stakeholder engagement and the initial literature review conducted to design the funding application. A realist review was completed as a first step to develop a programme theory from the international literature around co- and joint-responses for young people experiencing a mental health crisis (26). For the evaluation of the pilot, data will be collected at two time points (baseline within 12 weeks of first contact with emergency services and six-month follow-up) using quantitative surveys and semi-structured qualitative interviews. These widows were selected based upon feedback from practitioners and families, as the ‘crisis window' in terms of appointments, follow-up and care is often 10–12 weeks. A comparative approach will be taken, with participants recruited from both the Care Responders pilot and treatment-as-usual (TAU) emergency response pathways. TAU responses will provide context for understanding the pilot data collected. There is acknowledgement that TAU will be heterogeneous—this is of interest to the research team as the study is aiming to understand how similar or different the various TAU responses are to aid learning.

The evaluation will also include a nested case study in a neighbouring city to explore the delivery of an all-age 24/7 crisis service. Multiple perspectives will be sought from young people, parents/carers, practitioners, and service leads. Findings from these mixed data collection methods will be synthesised using a realist logic of analysis to further develop and test (confirm, refute or refine) the programme theory (from the realist review), and generate contextually grounded explanations of how, why, and for whom the Care Responders service intervention works. Findings will be used to inform recommendations for practice. The health economic cost consequence will be informed by the realist programme theory and, where possible, will test and refine theories in relation to economic evaluation, service resilience, and other key factors of importance identified.

### 2.4 Setting

The pilot and evaluation will be conducted in Greater Manchester (GM), a large metropolitan county in the Northwest of England with a population of approximately 2.9 million people. GM is the second most populous urban area in the United Kingdom after London and is made up of ten local authorities, including Manchester, Salford, and eight surrounding boroughs. The region is characterised by high levels of ethnic, cultural, and linguistic diversity, with over 200 languages spoken and nearly 40% of young people in Manchester identifying as multilingual. Alongside areas of significant economic growth and affluence, GM has some of the most deprived neighbourhoods in England; for example, 28% of children in Rochdale (a metropolitan borough with GM) live in poverty, compared to a national average of around 20% (27). Health inequalities are therefore a critical issue within the region, with mental health needs disproportionately affecting children and young people in lower-income communities.

### 2.5 Participant inclusion

Every person eligible to take part will be offered the same opportunities, regardless of any protected characteristics. Due to our aim to recruit children, young people and their parents/carers, our age range for the study is 5–99 years. Any young person who has accessed an emergency response to a mental health crisis (our Care Responders pilot and treatment as usual), and their parents/carers, will be eligible to take part. Data on protected characteristics (age, disability, gender reassignment, marriage and civil partnership, pregnancy and maternity, race, religion or belief, sex, and sexual orientation characteristics which are protected against discrimination under the UK Equality Act 2010) will be collected through standard demographic surveys from all participants and members of the research team, including stakeholders. Within our end of study reports, we will provide tabulated summaries of demographics of the research team, stakeholders, and participants to ensure transparency and accountability.

Across the study, up to a total of **360 participants** will be involved. This sample was informed by service activity data over a three-month period within one Greater Manchester borough scaled across the 10 Greater Manchester boroughs. The participants include:

- **Workstream 1**: Up to 40 professional stakeholders and/or people with lived experience of mental health crises (aged over 18).
- **Workstream 2**: Up to 100 children, young people and/or parents/caregivers supported by the Care Responders response; up to 100 children, young people and/or parents/caregivers who received usual care (TAU), and up to 60 practitioners from Greater Manchester. The care received by young people will be determined by availability at the time of the crisis, providing an opportunity to explore who may receive different types of support at different times.
- **Nested Case Study**: Up to 20 children and young people, up to 20 parents/caregivers, and up to 20 practitioners from across the case study site, who may be NHS practitioners and/or non-NHS community-based practitioners or stakeholders.

Demographic information will be collected from all participants, to ensure that transparency in reporting demographic information, and the critical appraisal of whether the inclusive research and engagement methods that we employ, invites people of all communities into the study. Demographics from NHS practitioners will be compared to NHS workforce data to undertake comparisons to explore representation. A similar process will be undertaken to explore how representative participant demographics are of the communities in which the Care Responders study is based.

### 2.6 Recruitment and consent procedures

Due to the varied nature of emergency care and the footprint of our study, there are five recruitment pathways for potential participants:

#### 2.6.1 Pathway one: care responders response

Young people and parents/carers who access support, via central triage, from the Care Responders project staff (police officer and mental health practitioner), will be recruited to the study via their engagement with the Care Responders response. Data will be collected as outlined in table one. Further details on consent for treatment and consent for evaluation participation are discussed in section ‘Informed Consent'.

#### 2.6.2 Pathway two: long-term treatment as usual

Young people and parents/carers who have accessed treatment as usual (TAU) over the last two years will need to hear about the study before they know whether they can participate in the evaluation, based on their engagement with an emergency response following a mental health crisis. Therefore, we will share information about the study through media, social media channels, information letters, and NHS webpages, sharing the study's designated email address and phone number, so people can contact us if they wish to opt-in and take part. Following an amendment to the original protocol, practitioners and their delegates in young people's NHS services will also gather consent to contact from eligible families, so the family do not need to take the first step to contact the research team.

#### 2.6.3 Pathway three: short-term usual care reporting

For potential participants who engage with TAU over the course of the study, we will ask attending first responders offering TAU to share a consent to contact form developed for this study with the parent/caregiver (not the young person) when they attend to young people experiencing a mental health crisis. Through the consent to contact form, parents/carers can opt-in to being contacted to hear more about the study. Young people will not be directly addressed about research participation at the time of the call out but, if parental/caregiver approval is in place to be contacted, the research team will be able to share information about the study with the young person at a later date to ask if the young person would like to be involved in the evaluation. If the young person has asked for information about the study, but consent to contact is not in place from the parent/caregiver, the parent/caregiver will be contacted to ask if their child (aged under 16 years old) can be provided with information about the study, in case the family wishes to support the young person to take part. Information flyers about the study and consent to contact forms will be shared by first responders and practitioners across the Northwest. Data will be collected as outlined in table one.

#### 2.6.4 Pathway four: nested case study

Information about the nested case study will be shared via practitioners, administrators, posters and leaflets across the 24/7 crisis service. Information will also be shared through local media and social media. Once in possession of the information about the study and contact details of the research team, potential participants will be able to opt-in. A member of the research team will then undertake a short eligibility check over the phone or by email, before the participant is consented into the study.

#### 2.6.5 Pathway five: participation via online qualtrics survey

In addition to the pathways outlined above, a fifth route to participation has been introduced following feedback that meeting with or speaking to a researcher in person about such a sensitive experience may not be manageable or preferable to participants. Concerns have been raised by clinical and voluntary and community sector enterprises (VCSE) colleagues around the potential for shame, stigma, and judgement associated with crisis experiences discouraging potential participants, as well as the additional emotional burden of recounting these events in a one-to-one research conversation so soon after the event.

To address these barriers and improve the acceptability and accessibility of participation, families will be given the option to complete a secure, self-administered online survey, hosted via Qualtrics (an online survey tool). Data will be collected as outlined in table one. Participants will be able to access the survey via a web link provided on study information materials distributed by first responders, NHS services, crisis response practitioners and the research team. The link will also be made available through the study's designated web page, NHS partner websites, and via social media posts from participating services.

This route offers participants a degree of anonymity and flexibility, allowing them to complete the survey at a time and location of their choosing, and at their own pace. This option removes the need for direct interaction with a researcher at the initial data collection stage, thereby creating a space in which young people and families may feel more able to reflect openly on their experiences without fear of judgement or perceived consequences. It also reduces the burden on families who may be managing multiple appointments and competing demands on their time.

For young people aged under 16, parental or caregiver consent to participate will be sought through the same process described in Pathway Three, with an option for parents/caregivers to consent for their child to complete the online survey independently where appropriate. Information about the online participation option will be included in all study recruitment materials to ensure families are aware of this additional, confidential, and flexible route for contributing to the evaluation.

Finally, on a case-by-case basis, if a young person does not feel comfortable to participate in person, over the phone, or may struggle to engage with the Qualtrics platform, a research worker will guide the participant to complete the steps of the Qualtrics based survey via email. This adjustment will need to be approved by the CI before being actioned and this adjustment will be recorded in the participant tracker to aid transparency.

#### 2.6.6 Feasibility risks and mitigation

Recruitment in acute and crisis settings present known challenges, particularly with vulnerable populations such as CYP in mental health crisis. Anticipated barriers include reluctance to revisit traumatic experiences, limited opportunity to approach individuals prior to discharge, and variability in engagement across recruitment pathways. To mitigate these risks, we will offer flexible options for participation, including the ability to engage at a later point post-crisis (within 12 weeks post crisis response, up to two years), and through different formats for sharing experiences. Recruitment will be supported via multiple pathways, including NHS services and partnerships with relevant VCSE organisations to maximise reach and ensure individuals can access the study at a time and in a manner that feels safe and acceptable to them.

The inclusion of multiple recruitment pathways represents a strength of the study in terms of accessibility and inclusivity. However, it also introduces a potential risk of selection bias, particularly between participants recruited through clinical pathways and those who self-selected via online methods. To address this, differences in recruitment routes will be explicitly considered during data interpretation and analysis. Reflexive analytic practices, informed by stakeholder engagement and communities of practice will be used to critically examine how recruitment context may shape participant perspectives. While offering open and flexible recruitment options aimed to mitigate exclusion, the implications of recruitment pathway differences will be kept under active consideration throughout the analytic process.

### 2.7 Participant support

Participants will be supported by the full research team, including the patient and public involvement and engagement (PPIE) lead (AT), the peer research assistant, and the Head of Patient and Carer Experience and Engagement at Pennine Care. If a participant wishes to withdraw from the study, a collaborative support plan will be developed to review options for their continued engagement. All young people will have access to the Pennine CAMHS mental health helpline and family support resources. Similar support will be offered to stakeholders, with signposting to external helplines. Staff participants will also be reminded of support services available through their employer, including those provided by the Trust. In recognition of participants' time and expertise, remuneration will follow INVOLVE guidance and has been detailed in the study budget.

#### 2.7.1 Safeguarding

The study involves potentially sensitive topics, and procedures are in place to recognise and respond to participant distress, in accordance with bespoke standard operating procedures and advisory distress protocols to manage any risk uncovered as part of the planned research data collection. These will comply with national and local policies for safeguarding children. The research includes a 12-week window post crisis to engage with the research, and an additional two year participation option for those preferring greater time post crisis, pausing or stopping data collection appointments, offering reassurance and breaks, reminding participants of their right to withdraw without detriment, and signposting to appropriate support or emergency services as required. All participants will receive debriefing information with details of local support services. All research staff will receive regular supervision and have access to clinically qualified members of the research team via an agreed clinical cover rota. Face-to-face contact will comply with lone working policies of participating NHS trusts and Higher Education Institutions in the UK.

### 2.8 Informed assent and consent

Informed consent and assent will be obtained in accordance with the existing NHS Standard Operating Procedure for a mental health joint response. For the intervention, verbal consent for joint police-mental health practitioner response is obtained during the 111/999 call. At the scene, verbal consent to engage with the evaluation is taken by the attending mental health practitioner and recorded in clinical documentation. The research team will then follow up to confirm written consent prior to any data collection.

For participants under 16 years, parental or caregiver consent will be required, with the young person providing assent where appropriate. Young people aged 16–18 years may provide their own consent, subject to the practitioner's assessment of capacity at the time. Given the potential distress during crisis call-outs, flexibility will be built into the process: information may be left for later consideration, or follow-up contact arranged once the young person or family can engage.

**Figure 1:**
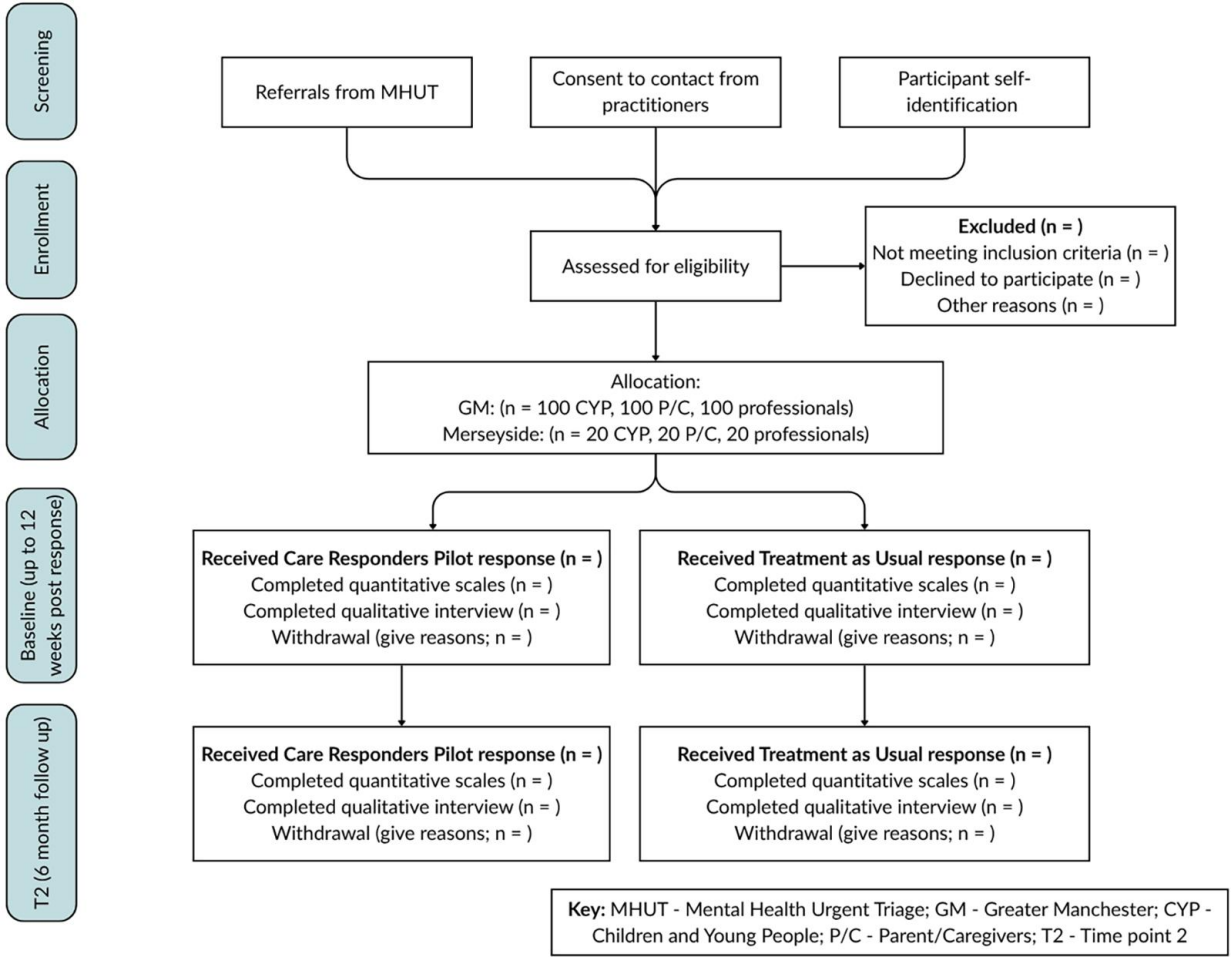
Participant flow through the study.

Consent will be documented through a range of options to maximise accessibility, including signed hard copies, electronic copies, audio-recorded consent, or remote confirmation. Assent/consent will be reviewed at each assessment point to ensure continued willingness to participate. Participants will also be offered the option to provide additional consent regarding use of anonymised data, recording for quality assurance or training, data sharing, follow-up for related research, and receipt of study findings. All participants will be free to withdraw at any time without consequence Figure 1.

### 2.9 Care responders intervention description

The Care Responders response protocol provides a structured, trauma-responsive framework for the Care Responders emergency mental health practitioners to children and young people aged 5–18 across Greater Manchester. Delivered jointly by two NHS mental health practitioners, funded by excess treatment costs, the practitioners work with officers and special constables of GMP to offer a joint response for young people. The Care Responders response aims to reduce restrictive interventions and unnecessary hospital conveyance by offering timely, community-based support. It outlines procedures for triage, risk assessment, and collaborative safety planning, with tailored guidance for different age groups and additional considerations for care-experienced, neurodiverse, disabled, and LGBTQIA + young people. Emphasising cultural sensitivity, caregiver engagement, and professional wellbeing, the protocol integrates evidence-based strategies to promote emotional safety, de-escalation, and recovery. It also supports the research and service evaluation through parallel clinical and research documentation processes. The Care Responders response protocol is supplemented by tailored self-care guides, co-produced with experts-by-experience. The guides are tailored by age groups and provide self-care information and outlines what support is available for future mental health difficulties. The guides are left with the family at the end of a Care Responders response.

The Care Responders operate across the 10 Greater Manchester boroughs associated with GMP. There are two pathways of referral to the care responders; through an emergency telephone call to the police or via a police officer notifying the practitioner that a minor is in custody. Referrals received are reviewed for eligibility in regard to the patient inclusion criteria and are prioritised based on the potential to manage the situation in the community.

Patient health records are reviewed along with police incident information including log number and reason for referral prior to assessment by mental health practitioners. For community referrals, a Care Responders practitioner will attend the scene supported by a special constable who will make sure that the scene is safe for the mental health assessment to take place. A special constable is not required for an assessment in the custody suite; however, a police officer remains close by for support. The mental health assessment includes information gathering on mental state examination and historic and current risk domains (risk to self, others, harm from others, vulnerability/neglect/exploitation). This information is used to develop a formulation regarding risk. During this assessment a safety management plan is completed with the young person and, if appropriate, responsible adult. Assessment and communication style are adapted dependant on the cultural, social and individual needs of the patient in line with the protocol. The appropriate self-care guides are provided to the young people and caregivers.

If appropriate, the research study is discussed and consent for contact is explored. Alternatively, information about the study is left with the young people and carers to be reviewed at a more convenient time.

Once an assessment is completed and recommendations are made, the Care Responders practitioner completes a General Practice (GP) letter outlining these recommendations including any referrals made. Urgent referrals for intensive crisis support are made to the crisis pathway via telephone up to 21:30. If a safety plan cannot be completed or the risk is unmanageable in the community, the patient would be redirected to A&E or their suitability for an alternative place of safety will be explored. Referrals for ongoing support which might include therapeutic intervention, neurodiversity assessment or medication review will be made to the appropriate service dependent on patients' locality and level of need. If a referral is rejected by a particular service, a follow up GP letter detailing the new recommendations will be provided. Clinical records are also added to the patients' care clinical records.

### 2.10 Data collection and management

Data will be collected at baseline (T1, within 12 weeks of first contact with emergency services) and at six-month follow-up (T2). Participants will complete quantitative measures and take part in semi-structured interviews exploring immediate experiences and subsequent reflections. Data will be gathered from young people and, where possible, their parents/carers. Quantitative measures were chosen to cover wellbeing and coping domains, and health economic data, along with the wide age range of CYP participants (5–18 years), thus tools were chosen which either had a broad age range, or different versions to meet the required age range. All study data will be handled in line with relevant national information governance legislation (e.g., GDPR) and NHS data governance standards, with identifiable information stored securely and separately from research data. Access will be restricted to authorised members of the research team, and all data will be anonymised or pseudonymised prior to analysis and dissemination Table 1, Table 2.

**Table 1:** Data collection points and tools.

Time point/Participant group	In person/video call T1 (within 12 weeks of engagement with Care Responders service/TAU)	In person/ video call T2 (6-month follow-up)	Qualtrics Survey T1 Route 1: within 12 weeks of engagement with Care Responders service/TAU Route 2: Within two years	Qualtrics Survey T2 (Only if Route 1: T1 was within 12 weeks of engagement with Care Responders service/TAU)
**Children/young people**	Personal context Nature of crisis Demographics SDQ—CYP (primary outcome measure) ESQ SRS SMFQ (primary outcome measure) CYRM-R KidCOPE Interview EQ-5D-Y	ESQ KidCOPE CYRM-R sMFQ (primary outcome measure) Interview/Workshop	Personal context Nature of crisis Demographics SDQ—CYP (primary outcome measure) ESQ SRS sMFQ (primary outcome measure) CYRM-R KidCOPE Interview questions EQ-5D-Y	Personal context Nature of crisis Demographics SDQ—CYP (primary outcome measure) sMFQ (primary outcome measure) CYRM-R KidCOPE Interview questions
**Parent/Carer**	Interview SDQ—Parent version (primary outcome measure) PSS STAR-P SRS EQ-5D	ESQ SDQ and PSS STAR-P EQ-5D Interview/Workshop	Interview questions SDQ—Parent version (primary outcome measure) PSS STAR-P SRS EQ-5D	Interview questions DQ—Parent version PSS
**First Responders/ Practitioners**	Demographics Interview STAR-C	Reflective interview in last two months of pilot. STAR-C	Demographics Interview STAR-C	N/A

**Table 2:** Explanation of tools used.

Measure (Acronym, full name)	Description
SDQ: Strengths and Difficulties Questionnaire (primary outcome measure)	A behavioural screening tool assessing emotional symptoms, conduct problems, hyperactivity/inattention, peer problems, and prosocial behaviour in children and young people (self- and parent-report versions).
ESQ: Experience of Service Questionnaire	A patient-reported experience measure assessing satisfaction with services, including perceived helpfulness and accessibility.
SRS: Session Rating Scale	A brief measure of therapeutic alliance evaluating the quality of the relationship between participant and practitioner, including agreement on goals and perceived support.
sMFQ: Short Mood and Feelings Questionnaire (primary outcome measure)	A self-report measure assessing depressive symptoms in children and young people over the past two weeks.
CYRM-R: Child and Youth Resilience Measure—Revised	A measure of resilience capturing individual, relational, and contextual factors that support coping with adversity.
KidCOPE Coping Measure	A brief self-report tool assessing coping strategies used by children and young people in response to stress or crisis.
EQ-5D-Y: EuroQol 5-Dimension Youth version	A standardised measure of health-related quality of life in children and young people across five domains: mobility, self-care, usual activities, pain/discomfort, and emotional wellbeing.
EQ-5D: EuroQol 5-Dimension	The adult version of the EQ-5D assessing health-related quality of life across the same five domains.
PSS: Perceived Stress Scale	A widely used measure assessing the extent to which individuals perceive their lives as stressful.
STAR-P: Scale to Assess Therapeutic Relationship—Parent version	A parent-reported measure of the therapeutic relationship, including collaboration, clinician input, and emotional bond.
STAR-C: Scale to Assess Therapeutic Relationship—Clinician version	A clinician-reported measure assessing the quality of the therapeutic relationship with service users.

Semi-structured interviews will be conducted with service users, parents/carers, and practitioners to explore experiences, acceptability, satisfaction, wellbeing, and implementation of the Care Responders pilot and TAU pathways. Interviews will take place at T1 and T2. Reflexive interviews will be offered to past-TAU participants and case study participants who had a crisis response within two years of participation of this study. Practitioners, including mental health professionals, police officers, and service leads, will also be interviewed throughout the study.

Participants may choose to take part in person (e.g., CAMHS, home, school, or workplace) or online via online via Microsoft (MS) Teams, and young people may invite a trusted adult to attend. Interviews (up to 90 min) will be audio-recorded, transcribed verbatim, anonymised, and analysed using a reflexive and transparent approach, with regular research team interpretation meetings. Topic guides will be iteratively refined throughout the study, and participants will be offered the opportunity to review transcripts and receive emerging findings to support member checking. At suitable points during the analysis process, anonymised sections of data and preliminary analysis will be discussed with the advisory groups of the study to ensure lived experience and stakeholder perspectives continue to inform programme theory development throughout.

### 2.11 Analysis plan

Initially, quantitative data will be entered into SPSS. Depending upon the completion of data sets and whether we achieve our recruitment targets, we will either conduct a descriptive analysis or regression analyses. Data analysis will be concurrent with data collection, in line with realist interviewing conventions (28). Data analysis will help us understand and explain why the Care Responders service works in the way it does for young people and families when called to a mental health crisis, in which contexts and to what extent. This will allow us to develop an in-depth, realist understanding and explanation of the impacts observed.

Rather than aiming for complete data across all measures, the study allows participants to self-select which components they wish to engage with, reflecting their interest and levels of comfort. This approach is intentional and aligned with the exploratory aims of the research, recognising that patterns of engagement and non-completion are themselves analytically meaningful. Quantitative analyses will therefore focus on descriptive and exploratory outcomes, with subgroup analyses undertaken only where data volume and completeness permit.

Each new element of relevant data will be used to further develop and test aspects of the programme theory. As it is developed and tested, data sources will be re-scrutinised to search for data relevant to the revised programme theory. Transcripts will be uploaded to NVivo (software that supports qualitative data analysis). Relevant sections of transcripts that have been interpreted as related to contexts, mechanisms and their relationships to outcomes will also inform our analysis. This coding will be both inductive (codes created to categorise data identified through the analysis process) and deductive (codes created in advance of data extraction and analysis as informed by the initial programme theory).

We will then use the realist logic of analysis (28–31) to develop context-mechanism-outcome-configurations (CMOCs) that bring together the different sources of data to provide causal explanations for outcomes of importance with our programme theory. In addition, we will apply a range of reasoning processes associated with realist analysis (32) to these data, such as juxtaposing data, unpicking conflicting data, and consolidating data, to explain why differences may arise across settings, and how and why identified outcomes have occurred (or not). Our ongoing application of a realist logic of analysis will be guided by a series of questions that members of the team have used in other realist projects:

1. Is this a piece of data that is relevant to programme theory development?
2. If so, do its contents provide data that may be interpreted as informing our understanding of a key aspect of our initial context, mechanism, outcome configurations (CMOCs)
3. For data that has been interpreted as functioning as context, mechanism or outcome, which CMOC does it belong to?
4. Are there further data to inform this particular CMOC—contained within this source or other sources? If so, which other sources?
5. How does this particular CMOC relate to others that have already been developed?
6. How does this particular CMOC relate to the programme theory?
7. In light of this particular CMOC and any supporting data, does the programme theory need to be changed?

We will then use this in-depth understanding and explanation as a starting point of our discussions with the stakeholder groups to refine the final theory.

To ensure active surveillance of harms, the research workers will also actively check for the occurrence of specific adverse events during the follow-up period. Participants will be offered flexibility regarding length of follow-up assessment meetings, including the option of having regular breaks and multiple, shorter testing sessions. To reduce the likelihood of missing data, a member of the research team will be able to make multiple attempts to contact participants to engage with aspects of the study up until the time a participant withdraws. Data can be gathered in person or over the phone, MS Teams, or by post. Spurious data will be discussed within the research team, who will decide upon an appropriate response (i.e., deletion, checking, repeated data collection).

### 2.12 Health economic data analysis

We will perform a cost-consequence analysis of the Care Responders service in addition to considering distributional impacts by age group, gender, sexual orientation, and ethnicity if data allows, in line with recently published Realist Economic Evaluation Methods (REEM) guidance, which details a way of assessing and comparing complex interventions. We will provide monetarised valuation of the effects of the programme and detail of who experiences them (younger person, young person's family, health service, police services). We will use a micro costing approach (33), using detailed data on resource utilisation from NHS Digital and unit cost data from the Personal Social Services Research Unit (PSSRU) to generate precise estimates of economic costs. Health and wellbeing outcomes can be monetarised through the calculation of healthy life expectancies and disability free life years (34, 35). Consequences will focus on the health and well-being impacts of the programme as well as impacts on the health service.

We choose a cost-consequence approach over other health economic evaluation techniques because this approach provides a detailed disaggregated presentation of multiple outcomes and costs side by side ensuring the granularity and complexity of the effects are not lost by condensing outcomes into a single metric such as QALYS. This approach is recommended for complex interventions when decision makers need to consider various factors—such as patient experience, NHS staff and police staff time and direct costs. Decision makers can apply their own priorities to the results (36).

We will consider the short-term cost and consequences of the Care Responders service using the estimated ex-poste effects from data collected in the quantitative surveys through Workstream 2. Additional consequences on outcomes related to changes in service usage and contact with the police using data from NHS Digital and the police will be obtained by estimating a quasi-experimental model such as difference-indifference or interrupted time series (37, 38). Ex-ante longer-term costs and consequences to young people and their families, health services, and police will be estimated using evidence from the review of the literature from Workstream 1. We will explore different time horizons given what data is available from the literature and discussions with stakeholders and the public. Discount rates of 3.5% will be used, as per guidelines (39).

The analysis will be conducted following well-established guidelines (39, 40). Missing data will be imputed. Subgroup analysis (distributional cost consequence analysis) will be conducted on samples large enough to identify any effects. Uncertainty will be incorporated using a combination of scenario based deterministic sensitivity analysis, threshold analysis, and/or probabilistic sensitivity analysis (34, 41). We will work with local partners to accurately audit the need and associated costs the Care Responders service could address, in terms of the numbers of referrals, call outs, and admissions to ED/s136s.

### 2.13 Public, patient and community involvement and engagement

A lived experience advisory group (*N* = 8) of young people (aged 16–25-years) and a parents and caregivers group (*N* = 8) have been appointed. In addition, an independent oversight group (*N* = 8) and an implementation advisory group (*N* = 12) have been established in line with NIHR guidance. Each group will meet every 3–6 months to ensure that their perspectives meaningfully shape the Care Responders study, which focuses on crisis care for children and young people.

Across these groups, members will contribute to:

- Realist interpretation of emerging findings
- Co-production of study materials, processes and recommendations
- Knowledge exchange and dissemination activities
- Guidance on implementation considerations to support real-world relevance and impact

These meetings will ensure that lived experience, caregiver perspectives, community insight, and independent scrutiny remain integral to the project throughout its duration. Additionally, a mobilisation steering group of service leaders will be established to support the development of the Care Responders service within the existing crisis infrastructure and its deployment within existing systems.

## 3 Expected outputs

The existing literature highlights a clear rationale for interventions that integrate services to combine strengths and resources. International guidance emphasises tiered, child-centred systems that combine early intervention with specialist care, alongside trauma-informed, formulation-led approaches that prioritise safety, relational trust, and the avoidance of re-traumatisation (6). However, persistent gaps in child- and crisis-specific mental health policy, coupled with variability in service delivery and workforce capacity, mean that many young people continue to encounter fragmented and risk-oriented responses, often involving police as default first responders. Frameworks such as MHPSS (9) further underline the importance of contextual, relationship-based care that promotes dignity, participation, and recovery. In this context, joint police-mental health interventions represent a potentially important mechanism for aligning real-world crisis responses with these principles by improving triage, reducing inappropriate criminal justice pathways, and supporting more collaborative, developmentally informed decision-making. Nonetheless, evidence that intervention effects are highly context-dependent reinforces the need for careful evaluation of both outcomes and implementation within UK systems such as the NHS.

The Care Responders study will produce a programme theory of co- and joint responses to mental health crisis for young people between mental health practitioners and police officers. The study aims to identify the mechanisms and contexts that influence outcomes, such as improved wellbeing, reduced hospital admissions, and age-appropriate trauma-informed crisis care. It will also explore the roles of professionals involved, assess cost-effectiveness through a health economic evaluation, and develop a realist programme theory to guide future service design. Outputs will include best practice tools, peer-reviewed publications, heat maps of need and service models, policy recommendations, anonymised datasets for future research, and creative dissemination activities such as animations and co-produced live performances. These findings will inform national guidance and support the development of more responsive, compassionate, and effective emergency mental health services for young people.

## 4 Ethics Statement

Ethical approval was sought from the Health Research Authority (HRA) through the Greater Manchester Research Ethics Committee (REC; ID 332304) and local site approvals were then sought. Over the course of the first year, a series of stakeholder interviews and meetings were held, which informed further refinement of the research protocol and mechanisms for recruitment and participant support (see Supplementary Material File 2 for a table explaining the feedback and how we implemented changes via amendments). All but one of the amendments was approved by the NHS sponsor. The use of Snapchat as an alternative means of communicating with young people via the dedicated study NHS phone. The team applied for approval to use Snapchat as email and SMS are not typical platforms for communication used by young people. The security settings on Snapchat are similar to SMS. The study team developed a protocol for the use of Snapchat, which was approved by the HRA and the Research and Innovation department of the Sponsor, but was rejected by the Information Governance team of the Sponsor and so is not in use.

## 5 Dissemination plan

Findings will be disseminated through peer-reviewed publications, conferences, public forums, and co-produced live performances with Made by Mortals (an immersive theatre group). The immersive theatre productions will aid our testing and refinement of the programme theory post data-collection. Participants will receive accessible summaries of results, and a multimedia portfolio will be co-produced with lived experience advisors and stakeholders for public dissemination.

Made By Mortals will produce content for up to three live performances towards the end of the study. The performance will promote the benefits and impact of the new approach explored within the research. The performance will be coproduced by young people (aged 16–25-years) with lived experience of crisis care as well as mental health practitioners, and police (involved in the study), and family members. The performance will bring ‘real people's' lived experiences into stark reality, the real-life experience of people who have lived through and been impacted by Crisis to support policymakers and other stakeholders to understand the human impact of the new approach.

Through an interactive workshop, it will also give them knowledge and space needed to consider the changes and commitments they need to make in-order to implement the new approach into their systems.

Made by Mortals have their own process for gaining informed consent from people engaging in their productions as a participatory arts organisation. Where people opt-in to solely take part in the co-production and performance process, they will follow the Made by Mortals consent process. Participants of the study who are aged 16-years and over will also have the option to check the box on their consent form for the research study to hear about the Made by Mortals project within the study. If they decide to get involved, they will then follow the Made by Mortals process for providing their consent. Made by Mortals are highly experienced in working with young people in relation to mental health narratives and have a variety of engagement options available to promote choice within the development process.

Made By Mortals will provide all creative and technical staff to deliver the performances. They will also produce social media assets, photographs, and blogs to promote the project. Made By Mortals will produce a shorter presentation-style performance for conference events to support dissemination. Co-production workshops can be delivered in-person or online to best meet the needs of the lived experience groups. Made By Mortals will make payments to stakeholders for their contribution, as per INVOLVE Guidelines.

The development and dissemination of the Care Responders best practice tools will play a central role in influencing wider policy and practice across children and young people's crisis care. These tools, co-produced with young people, families, practitioners, and system leaders, based on the findings of the study, will translate the programme theory and evaluation findings into actionable guidance for commissioning, workforce development, and cross-agency crisis pathways. To support system-level change, the research team will engage regional and national policy teams (including NHS England, Integrated Care Boards, and policing/health liaison forums) through targeted knowledge-exchange events, policy briefings, and stakeholder workshops. By aligning the tools with existing national priorities for crisis transformation and integrated care, we anticipate they will inform future service models, support scale-up of joint response approaches, and shape guidance on age-appropriate, trauma-informed emergency mental health care for children and young people.
