# Supplementary material for: Evaluating the implementation of a mental health joint response with young people and families: protocol for the care responders study, a realist and health economic evaluation
Source: Front Health Serv. 2026 Jun 26;6:1846383. doi: 10.3389/frhs.2026.1846383 (PMC13350323; doi:10.3389/frhs.2026.1846383)
Supplement: Supplementary file 1 [file Table1.docx]

**Supplementary file 1**

**SPIRIT 2013 Checklist**

1. Administrative Information

| Section | SPIRIT Item | Description | Where Addressed in Protocol |
| --- | --- | --- | --- |
| Title | 1 | Descriptive study title identifying intervention and population | Title page; Abstract |
| Trial registration | 2a | Trial identifier and registry | “ISRCTN10170634” in Abstract |
|  | 2b | All WHO items included in registration | Registry number listed |
| Protocol version | 3 | Date/version identifier | File name v2; internal comments indicate versioning |
| Funding | 4 | Sources and types of support | “Funding: NIHR HS&DR (£953,791)…” |
| Roles & responsibilities | 5a | Contributors and roles | “Author Contributions” section |
|  | 5b | Sponsor name & contact | NHS Sponsor described in Ethics section |
|  | 5c | Committees (steering, advisory) | Lived experience groups; oversight group; implementation group |

2. Introduction

| Section | SPIRIT Item | Description | Where Addressed |
| --- | --- | --- | --- |
| Background & rationale | 6a | Description of research question & justification | “Background & Rationale” |
| Objectives | 7 | Specific aims and hypotheses | “Aims” and “Objectives” |
| Trial design | 8 | Description of design, allocation, framework | “Design” — realist evaluation; mixed-methods; comparative; nested case study |

3. Methods: Participants, Interventions, and Outcomes

Trial Setting

| Item | Description | Where Found |
| --- | --- | --- |
| 9 | Description of study settings | “Setting” (Greater Manchester; 24/7 site etc.) |

Eligibility Criteria

| Item | Description | Location |
| --- | --- | --- |
| 10 | Inclusion/exclusion criteria | “Participant Inclusion” section |

Interventions

| Item | Description | Location |
| --- | --- | --- |
| 11a | Detailed description of intervention | “Care Responders Intervention Description” |
| 11b | Criteria for discontinuation | Not harmful; community-based; redirection to A&E if risk unmanageable |
| 11c | Strategies to improve adherence | Practitioner training; protocol; PPIE; monitoring |
| 11d | Concomitant care allowed | TAU crisis pathways described |

Outcomes

| Item | Description | Location |
| --- | --- | --- |
| 12 | Primary & secondary outcomes | Quantitative measures table (SDQ, sMFQ, CYRM-R, etc.); CMOCs for realist outcomes; health economics measures |

4. Methods: Assignment of Interventions (Allocation)

*Note: The study is non‑randomised and comparative. SPIRIT items requiring randomisation are marked Not Applicable.*

| Item | Description | Status |
| --- | --- | --- |
| 16a | Sequence generation | Not applicable (no randomisation) |
| 16b | Allocation concealment | Not applicable |
| 16c | Implementation | Not applicable |

Blinding

| Item | Description | Status |
| --- | --- | --- |
| 17a | Blinding of participants/personnel | Not blinded — realist evaluation, open-label |
| 17b | Circumstances for unblinding | Not applicable |

5. Data Collection, Management, & Analysis

Data Collection Methods

| Item | Description | Location |
| --- | --- | --- |
| 18a | Data collection plans | “Data Collection and Management”; Table One |
| 18b | Participant retention | Follow-up flexibility; multiple contact attempts; multi-modal completion |

Data Management

| Item | Description | Location |
| --- | --- | --- |
| 19 | Data entry, coding, security, storage | GDPR/NHS IG standards in “Data Collection and Management” |

Statistical / Analytical Methods

| Item | Description | Location |
| --- | --- | --- |
| 20a | Statistical analysis | “Analysis Plan” |
| 20b | Additional analyses | Realist CMOC refinement; health economic sensitivity analysis |
| 20c | Definition of analysis population | “Participant Inclusion” and recruitment pathways |

6. Methods: Monitoring

| Item | Description | Location |
| --- | --- | --- |
| 21a | Data Monitoring Committee | Not required for low-risk evaluation; oversight groups used |
| 21b | Interim analyses | Not applicable |
| 22 | Harms | “Active surveillance of harms” |
| 23 | Auditing | Sponsor oversight via HRA and R&I processes |

7. Ethics & Dissemination

| Item | Description | Location |
| --- | --- | --- |
| 24 | Research ethics approval | “Ethics Statement” (REC ID 332304) |
| 25 | Protocol amendments | Described under ethics (Snapchat amendment example) |
| 26a | Consent process | “Informed Assent and Consent” section |
| 26b | Additional consent provisions | For data sharing, recordings, future contact |
| 27 | Confidentiality | GDPR-compliant storage, anonymisation |
| 28 | Declaration of interest | “Conflict of Interest” |
| 29 | Data access | Not explicitly stated — recommend adding (I can draft text if helpful) |
| 30 | Ancillary/post-trial care | Support via CAMHS helplines; stakeholder signposting |
| 31a | Dissemination policy | “Dissemination Plan” |
| 31b | Authorship eligibility | Covered under “Author Contributions” |
| 31c | Public access to protocol/data | Planned anonymised datasets for future research |

**CONSORT 2010 Checklist — Care Responders Study**

| Section / Topic | Item No. | CONSORT Checklist Item | Reported in Protocol |
| --- | --- | --- | --- |
| Title & Abstract | 1a | Identification as a randomised trial in title | N/A – non-randomised realist evaluation |
| Title & Abstract | 1b | Structured summary of design, methods, results, conclusions | Abstract provides structured background, methods, results, discussion |
| Introduction | 2a | Scientific background & explanation of rationale | Background & Rationale section |
| Introduction | 2b | Specific objectives or hypotheses | Aims and Objectives |
| Methods – Trial Design | 3a | Description of trial design | Realist, mixed‑methods, comparative, nested case study |
| Methods – Trial Design | 3b | Changes to methods after start | Amendments described in Ethics section |
| Participants | 4a | Eligibility criteria | Participant Inclusion section |
| Participants | 4b | Settings and locations | Greater Manchester & case study site |
| Interventions | 5 | Description of interventions | Care Responders Intervention; TAU pathways |
| Outcomes | 6a | Pre-specified outcomes | Table One; Realist CMOCs; economic outcomes |
| Outcomes | 6b | Changes to outcomes | None reported |
| Sample Size | 7a | How sample size was determined | Feasibility-driven; up to 370 participants |
| Sample Size | 7b | Interim analyses | N/A |
| Blinding | 11a | Blinding of participants/personnel | Open-label realist evaluation |
| Blinding | 11b | Blinding of outcome assessment | Not blinded |
| Statistical Methods | 12a | Methods to compare groups | Descriptive or regression analyses |
| Statistical Methods | 12b | Additional analyses | Realist CMOCs; cost-consequence analysis |
| Results – Flow | 13a | Participant flow | Provided in supplementary table |
| Results – Flow | 13b | Losses/exclusions | Tracked (crisis, safeguarding) |
| Recruitment | 14a | Recruitment dates | T1 within 12 weeks; T2 at 6 months |
| Recruitment | 14b | Why trial ended or stopped | Not applicable yet |
| Other Information | 23 | Registration number | ISRCTN10170634 |
| Other Information | 24 | Protocol access | Full protocol available |
| Other Information | 25 | Funding | NIHR HS&DR + excess treatment costs |
| Other Information | 26 | Competing interests | None declared |
