# Supplementary material for: Evaluating the implementation of a mental health joint response with young people and families: protocol for the care responders study, a realist and health economic evaluation
Source: Front Health Serv. 2026 Jun 26;6:1846383. doi: 10.3389/frhs.2026.1846383 (PMC13350323; doi:10.3389/frhs.2026.1846383)
Supplement: Supplementary file 2 [file Table2.docx]

Supplementary material 2: Implementation of stakeholder feedback via study amendments

| **Feedback** | **Rationale** | **Amendment** |
| --- | --- | --- |
| 1. Recruitment flyers could be more young people focused and clear | To enhance accessibility and make the recruitment flyers more visually engaging in the hope of promoting recruitment | 2 new recruitment flyers made with youth appropriate imaging and wording |
|  | To ensure the questionnaire remained relevant and captured the needs of the research but also to make it easier and less time consuming for the participant | Health economic questionnaire developed |
| 1. Make recruitment clearer | To enhance accessibility for a wider audience. To appeal to CYP in the hope of enhancing recruitment | Recruitment video developed |
| 1. Reduce wording on PIS for young people | To ensure age-appropriate materials were provided for all YP interested in participation | 5-15 and 16-18 TAU information sheets developed |
| 1. Additional recruitment options for nested case study site | To enhance and facilitate engagement in response to site challenges with recruitment | Focus group option included for nested case study |
|  | Administrative update for the purpose of the research team | Placeholders included instead of specific dates and versions for consent forms in case of other amendments of PIS |
| 1. Inauthentic participants were coming forward | To ensure that authentic participants are recruited in line with University of Manchester guidance | Inclusion of information about inauthentic participants into PISs |
| 1. Option needed for NHS to consent to contact to aid recruitment via clinical services | Feedback from YPs and practitioners in CAMHS which stated that interested participants might prefer to be contacted by the research team instead of them having to initiate contact. To do so, a consent to contact option is required. | Consent to contact form |
|  | Based on recent amendments, the protocol was required to be updated | Protocol updated reflected amendments to pathways |
| 1. Participants requested alternative options for involvement, worrying about potential stigma of discussing experiences to a researcher. Young people requested snapchat as an option for initial contact as don't want to share phone number/email | Additional communication method for initial contact to enhance participation | Inclusion of an online survey, inclusion of snapchat as an initial contact method (PIS, consent forms changed as a result) |
|  | To support trainee clinical psychologist thesis projects | Trainee's indicative topic guides for interviews (GPs and police staff focused) |
| 1. Young people and parents-caregivers may not always feel they have the words to explain their experiences of mental health crisis support | Alternative options for engagement in the study to aid participation options | Inclusion of an upload option on the survey for participants to upload creative submissions (e.g., poems, artwork, voicenotes, reflections etc) that represent their experiences |
